# COVID-19 and athletes: Endurance sport and activity resilience study—CAESAR study

**DOI:** 10.3389/fphys.2022.1078763

**Published:** 2022-12-16

**Authors:** Daniel Śliż, Szczepan Wiecha, Katarzyna Ulaszewska, Jakub S. Gąsior, Marcin Lewandowski, Przemysław Seweryn Kasiak, Artur Mamcarz

**Affiliations:** ^1^ 3rd Department of Internal Diseases and Cardiology, Medical University of Warsaw, Warsaw, Poland; ^2^ Students’ Scientific Group of Lifestyle Medicine, 3rd Department of Internal Medicine and Cardiology, Medical University of Warsaw, Warsaw, Poland; ^3^ Polish Society of Lifestyle Medicine, Warsaw, Poland; ^4^ School of Public Health, Postgraduate Medical Education Center, Warsaw, Poland; ^5^ Department of Physical Education and Health in Biala Podlaska, Faculty in Biala Podlaska, Jozef Pilsudski University of Physical Education in Warsaw, Biala Podlaska, Poland; ^6^ Department of Pediatric Cardiology and General Pediatrics, Medical University of Warsaw, Warsaw, Poland; ^7^ Department of Pharmacology and Clinical Pharmacology Collegium Medicum, Cardinal Stefan Wyszyński University in Warsaw, Warsaw, Poland

**Keywords:** physical activity, CPET cardiopulmonary exercise testing, VO_2max_ (maximal oxygen uptake), endurance athlete, SARS-CoV-2, COVID-19

## Abstract

**Background:** The COVID-19 pandemic and imposed restrictions influenced athletic societies, although current knowledge about mild COVID-19 consequences on cardiopulmonary and physiologic parameters remains inconclusive. This study aimed to assess the impact of mild COVID-19 inflection on cardiopulmonary exercise test (CPET) performance among endurance athletes (EA) with varied fitness level.

**Materials and Methods:** 49 EA (n_male_ = 43, n_female_ = 6, mean age = 39.94 ± 7.80 yr, height = 178.45 cm, weight = 76.62 kg; BMI = 24.03 kgm^−2^) underwent double treadmill or cycle ergometer CPET and body analysis (BA) pre- and post-mild COVID-19 infection. Mild infection was defined as: (1) without hospitalization and (2) without prolonged health complications lasting for >14 days. Speed, power, heart rate (HR), oxygen uptake (VO_2_), pulmonary ventilation, blood lactate concentration (at the anaerobic threshold (AT)), respiratory compensation point (RCP), and maximum exertion were measured before and after COVID-19 infection. Pearson’s and Spearman’s r correlation coefficients and Student t-test were applied to assess relationship between physiologic or exercise variables and time.

**Results:** The anthropometric measurements did not differ significantly before and after COVID-19. There was a significant reduction in VO_2_ at the AT and RCP (both *p* < 0.001). Pre-COVID-19 VO_2_ was 34.97 ± 6.43 ml kg·min^−1^, 43.88 ± 7.31 ml kg·min^−1^ and 47.81 ± 7.81 ml kg·min^−1^ respectively for AT, RCP and maximal and post-COVID-19 VO_2_ was 32.35 ± 5.93 ml kg·min^−1^, 40.49 ± 6.63 ml kg·min^−1^ and 44.97 ± 7.00 ml kg·min^−1^ respectively for AT, RCP and maximal. Differences of HR at AT (*p* < 0.001) and RCP (*p* < 0.001) was observed. The HR before infection was 145.08 ± 10.82 bpm for AT and 168.78 ± 9.01 bpm for RCP and HR after infection was 141.12 ± 9.99 bpm for AT and 165.14 ± 9.74 bpm for RCP. Time-adjusted measures showed significance for body fat (r = 0.46, *p* < 0.001), fat mass (r = 0.33, *p* = 0.020), cycling power at the AT (r = −0.29, *p* = 0.045), and HR at RCP (r = −0.30, *p* = 0.036).

**Conclusion:** A mild COVID-19 infection resulted in a decrease in EA’s CPET performance. The most significant changes were observed for VO_2_ and HR. Medical Professionals and Training Specialists should be aware of the consequences of a mild COVID-19 infection in order to recommend optimal therapeutic methods and properly adjust the intensity of training.

## Introduction

It was calculated that 18.2 million people died as a result of the pandemic between 2020 and 2021 and numerous deaths were due to coronavirus disease 2019 (COVID-19) (Wang et al., 2022). The numbers were influenced by limited prevention and treatment measures aimed at other diseases. In the case of convalescents, persistent symptoms are common after the infection (Nasserie et al., 2021). To mention, one of the most important health consequences of COVID-19 infection are CT changes in the respiratory system, primarily lunges (Baratella et al., 2021). Although, physical activity prior COVID-19 might alleviate severity and reduce mortality range in COVID-19 patients. It is especially recommended to include physical activity in the amount of ≥150 min/week of moderate activity or ≥75 min/week of vigorous activity (Sittichai et al., 2022). Even full vaccination does not guarantee complete protection against long-lasting COVID-19 complications (Al-Aly et al., 2022).

The period of pandemic and the anxiety associated with it made a huge impact on the lifestyle of people around the world (Jodczyk et al., 2021; Jodczyk et al., 2022). Many areas of human life were negatively affected, including mental health (Gruba et al., 2021; Jodczyk et al., 2022). Depression, anxiety disorders, sleep disturbances, and perceived stress became more common compared to the period before COVID-19, both among healthy young individuals and those yet suffering from any diseases (Kasiak et al., 2022a). The shift in everyday routine also elicited reports of decreased physical activity in different populations (Wunsch et al., 2022).

New, pandemic conditions became a challenge for sportsmen. Although, athletes are not considered a high-risk group for high COVID-19 vaccine hesitancy (Ulaszewska et al., 2022). Moreover, about 94% of them, the course of the illness is asymptomatic or mild (Lemes et al., 2022). Nevertheless, the return to physical activity after the disease raised questions in terms of safety with myocardial involvement as the main concern (Rajpal et al., 2021; Modica et al., 2022). Health hazards other than cardiovascular sequelae include respiratory (Xia et al., 2020) or muscular (Seixas et al., 2022) complications. Although, these health complications do not always require hospitalization. Moreover, training in confined areas (Washif et al., 2022) and sports events (Sparrow et al., 2021) encountered significant organizational problems due to the risk of SARS-CoV-2 transmission.

The above-mentioned factors remain closely related to physical performance. There is evidence of reduced oxygen uptake (VO_2_) and altered lactate metabolism in athletic convalescents after mild COVID-19. This issue is still not enough studied, but the physical capacity of the subjects tended to decline (Csulak et al., 2021; Komici et al., 2021). There is also a study whose results deviate from this assumption (Fikenzer et al., 2021). Cardiopulmonary exercise testing (CPET) has proven to be a useful tool in evaluating endurance athletes after COVID-19 and could be used to diagnose persisting symptoms (Moulson et al., 2022).

We stipulate that COVID-19 infection causes a deterioration in fitness performance with the aggravation of the body’s physiology and anthropometric measures (Ali and Kunugi, 2021), changes are noted especially in muscle building, the mechanism is not clear, but it may be due to e.g., the cytokine storm, malnutrition, prolonged inactivity (Zhou et al., 2020). This study aimed to: (1) assess COVID-19 consequences among endurance athletes with varied fitness level on exercise and body physiology and (2) adjust obtained results to time passed from COVID-19 infection. The main novelty and advantage of the present study is providing time adjusted results of CPET performance and somatic changes depending on the time that has passed since the COVID-19 infection. This approach enable to better understand possible consequences of the underwent disease. Moreover, we included EA with varied level of advancement, both professional and amateur ones, which facilitate more holistic and comprehensive assessment of COVID-19 infection on CPET performance.

## Materials and methods

### Study sample and testing protocol

This study was carried out on a group of young endurance athletes from June 2021 to June 2022 at the Tertiary Care Centre Sports Medicine Clinic (www.sportslab.pl; accessed on 2 February 2022, Warsaw, Poland). The recruitment process involved only endurance athletes who underwent a CPET performed pre and post-COVID-19 infection. Each participant signed informed consent. Study sample consisted of individuals with varied fitness level, both professional and amateur EAs.

**FIGURE 1:**
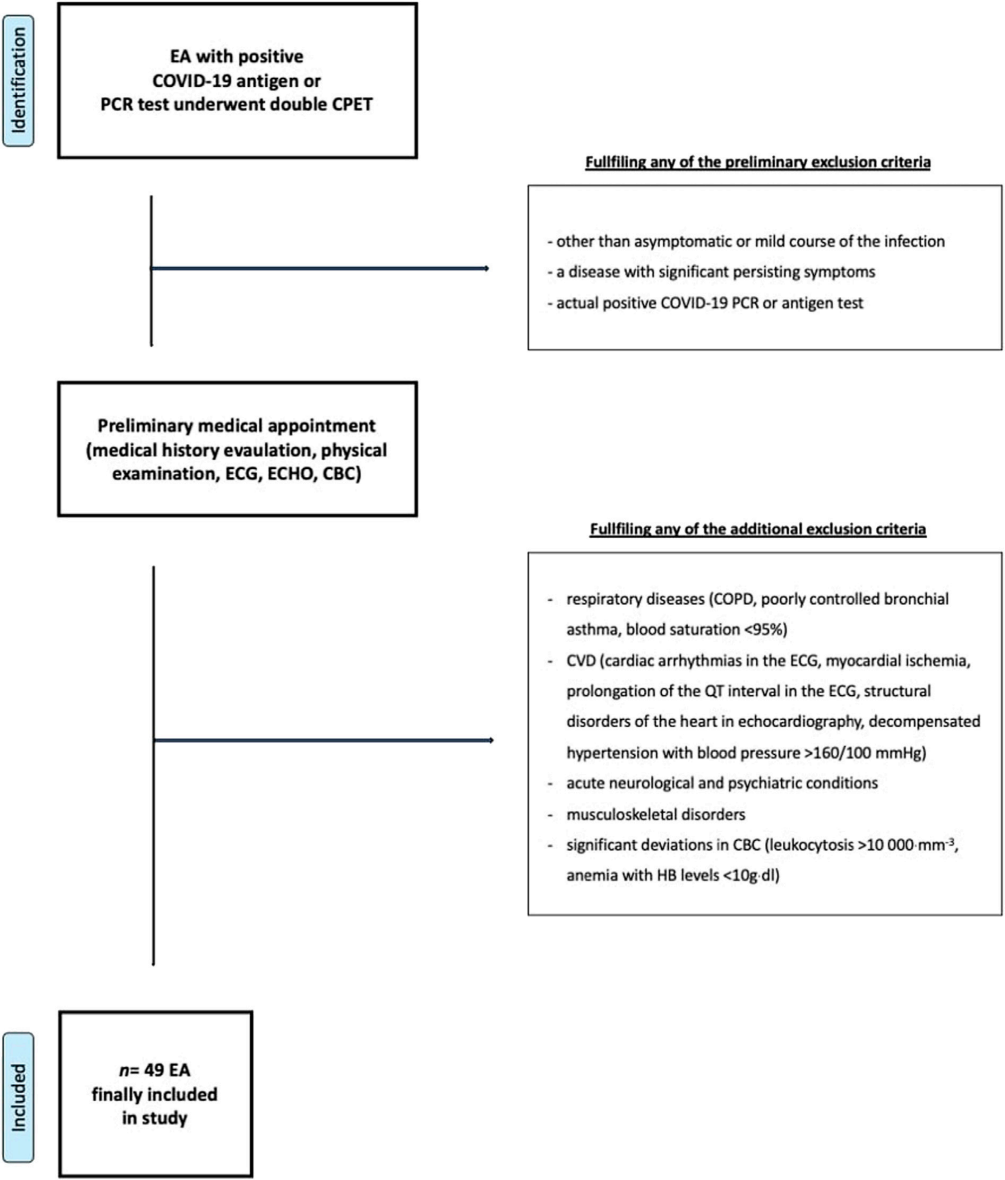
Participants selection protocol. Abbreviations: EA, endurance athlete; CPET, cardiopulmonary exercise test; ECG, 12-lead electrocardiogram; CBC, complete blood count; COPD, Chronic obstructive pulmonary disease; HB, blood hemoglobin.

The participants’ selection process has been shown in Figure 1. The inclusion criteria included: treadmill or cycle ergometer CPET in the 3 years before SARS-CoV-2 inflection, SARS-CoV-2 infection confirmed by PCR or antigen test, lack of hospitalization in a period from 2 weeks up to 6 months before post-COVID-19 CPET, asymptomatic or mild course of the disease with no persisting symptoms for >14 days, actual negative SARS-CoV-2 PCR or antigen test. The exclusion criteria included: respiratory diseases (COPD, poorly controlled bronchial asthma, blood saturation <95%), cardiovascular diseases (cardiac arrhythmias confirmed by ECG, myocardial ischemia, prolongation of the QT interval in the ECG, any structural disorders of the heart found in echocardiography, decompensated hypertension with blood pressure over 160/100 mmHg), acute neurological and psychiatric conditions, acute or chronic musculoskeletal condition, deviations in laboratory tests (leukocytosis over 10,000 mm^−3^, anemia with hemoglobin levels <10 g·dL^−1^dl).

Participants had a pre-CPET medical appointment with the cardiologist. A medical professional examined their past medical history and performed a physical examination, supplemented with a 12-lead ECG and echocardiography. Only athletes without cardiac or pulmonary abnormalities were finally qualified for the study.

43 males and 6 females fulfilled all inclusion criteria. Athletes underwent a treadmill or cycle ergometer CPET (a protocol and machine identical to the one used before the SARS-CoV-2 infection).

### CPET protocol

Body composition analysis (BA) was analyzed with a Tanita device (Tanita, MC 718, Japan) prior to each CPET with multifrequency 5 kHz/50 kHz/250 kHz. BA and CPET were conducted under the same conditions: 40 m^2^ air-conditioned area, altitude 100 m, MSL temperature 20–22°C, humidity 40–60%. Participants received dietary and recovery recommendations *via* e-mail prior to the CPET to be prepared well. Cycle CPET was performed on Cyclus-2 (RBM elektronik-automation GmbH, Leipzig, Germany), while treadmill on a mechanical treadmill (h/p/Cosmos quasar, Germany). Post-COVID-19 CPET was on the same modality as the pre-COVID-19. Cardio-pulmonary indices were measured *via* Cosmed Quark CPET device (Rome, Italy), and adjusted individually before each test (as recommended by the manufacturer’s instructions). HR was measured with the usage of the ANT + chest strap (as part of the Cosmed Quark CPET set) with accuracy comparable to ECG, ±1 bpm. For each athlete, the starting power (Watt) or speed (km/h) was individually determined. The starting power for the cycle ergometer CPET was the lowest value at which the participant declared resistance. For the treadmill, the initial speed was a ‚conversation’ pace. The test began with 5 min warm-up (walking or pedaling without resistance). The velocity was raised each 2 min by 1 km/h or the power by 20–30 W. For the treadmill CPET inclination was set at 1%. To obtain the maximum level of symptom-free exertion, sportsmen were instructed to maintain the effort as long as possible. They could end the protocol at any moment if felt they had reached their maximum. To ensure that participants achieved maximal effort, only those who’s met ≥4 the following criteria were included: (1) RER ≥1.10, (2) presented VO_2_ plateau (a VO_2_ growth <100 ml min^−1^ with increasing speed/power), (3) f_R_ >45 min^−1^, and (4) declared exertion ≥18 according to the Borg scale, (5) highest achieved HR **≤** 15 bpm under predicted HR_max_ (Lach et al., 2021). The exercises were terminated externally if VO_2_ or HR no further increase with increasing speed or power. CPET measures were by breath-by-breath method. VO_2max_ was averaged across 10-s intervals directly before termination. The peak HR was used in the analysis and the HR values were not averaged. Blood lactate was measured by obtaining 20 μ L blood sample from the fingertip. The measures were conducted before the test, after intensity change and 3 min after termination and analyzed in Super GL2 analyzer (Muller Geratebau 157 GmbH, Freital, Germany). First drops were taken into a swab before the proper sample has been taken. AT was considered after fulfilling all the following criteria: (1) VE/VO_2_ curve increased with the stable VE/VCO_2_ curve and (2) the end-tidal O_2_ partial pressure increase with the stable end-tidal CO_2_ partial pressure. RCP was considered after fulfilling all the following criteria: (1) a decrease in end-tidal CO_2_ partial pressure after achieving a maximal exertion; (2) a rapid increase in VE (second deflection); (3) the VE/VCO_2_ proportion meets the lowest value and begin to increase, and (4) a increase in VCO_2_ compared to VO_2_ (divergence from linearity).

### Statistical analysis

Basic participants’ data were anonymized, exported, and saved as an Excel file (Microsoft Corporation, Washington, DC, United States. The Shapiro-Wilk test was performed to assess normality. All variables were considered as continuous and calculated as means with standard derivation (SD). For normally distributed data Pearson’s r correlation coefficient was applied and for non-normally distributed variables the Spearman rank correlation coefficient was used. To compare means between the most significant variables adjusted for training experience, additional Student t-test for independent means heave been performed. The significance borderline was set at *p* = 0.05. Analyses were performed with the usage of statistical software SPSS Statistics (version 28, IBM, Chicago, IL, United States) and STATISTICA (version 13.3, StatSoft Polska Sp.z. o.o., Kraków, Poland).

### Ethics

This study was approved by the Bioethics Committee of the Medical University of Warsaw (approval no. KB/50/21 from 19th April 2021). Study procedures were in line with the declaration of Helsinki. Each athlete got detailed study information and signed informed consent before participating in the protocol.

## Results

### Participants basic characteristics

The characteristics of the population, including data such as height, weight, BMI, fat-free mass), BF (body fat), and FATM (fat mass), are presented in Table 1. Mean age during post-COVID-19 evaluation was 39.94 ± 7.80 years (40.74 ± 6.98 years for males and 38.09 ± 6.43 for females). We noticed the weight and BMI increases. Interestingly, the percentage of BF and FATM is lower after contracting COVID-19.

**TABLE 1:** Participants characteristics.

Variable			Males (*n* = 43)	Females (*n* = 6)
Age			40.74 ± 6.98	38.09 ± 6.43
Height			178.45 ±a 6.81	178.39 ± 6.85
CPET modality	Treadmill		25 (51.02%)	4 (8.16%)
	Cycle ergometer		16 (32.65%)	2 (4.08%)
Interval between CPET			591.67 ± 282.24	
Interval between pre-COVID-19 CPET and negative PCR test (end of COVID-19)			436.40 ± 290.40	
Interval between post-COVID-19 CPET and negative PCR test (end of COVID-19)			155.27 ± 82.52	
Primary sport discipline		Running	16 (32.65%)	4 (8.16%)
		Cycling	13 (26.53%)	1 (2.04%)
		Other	14 (28.57%)	1 (2.04%)
Waived competition due to COVID-19 infection		Yes	21 (42.86%)	2 (4.08%)
		No	23 (46.94%)	4 (8.16%)
		Pre-COVID-19	Post-COVID-19	*p*-value
Weight		76.62 ± 10.02	76.66 ± 10.90	0.951
BMI		24.03 ± 2.49	24.04 ± 2.71	0.931
FFM		63.36 ± 7.63	63.45 ± 7.97	0.774
BF		17.09 ± 4.73	16.91 ± 5.12	0.604
FATM		13.27 ± 4.68	13.20 ± 5.23	0.848

Abbreviations: COVID-19, coronavirus disease 2019; BMI, body mass index (kg·m^-2^); FFM, fat-free mass (kg); BF, body fat (%); FATM, fat mass (kg). Age is presented in years. Heigh is presented in cm. Weight is presented in kg. Intervals between CPETs is presented in days. Intervals between pre- and post-COVID-19 CPETs and PCR are presented in days

Data are presented as means with standard derivation (±) for continuous variables and number with percentage (%) for categorical variables.

The anthropometric measurements did not differ substantially before and after COVID-19. Meanwhile, there was a significant change in CPET performance. Both VO_2_ at the anaerobic threshold (VO_2AT_, *p* < 0.00001) and VO_2_ at the respiratory compensation point (VO_2RCP_, *p* < 0.00001) differ between pre- and post-infection conditions. Relative and absolute values were lower during the post-infection assessment. Heart rate at the anaerobic threshold (HR_AT_, *p* = 0.00140) and heart rate at the respiratory compensation point (HR_RCP_, *p* = 0.00011) were also aggravated and higher values were noted for post-infection measurement. Whereas lactate concentration and pulmonary ventilation (VE) were decreased only at the respiratory compensation point (Lac_RCP_, *p* = 0.01250, and VE_RCP_, *p* < 0.00001). The maximal oxygen uptake (VO_2max_) was also diminished in the post-COVID-19 period (*p* = 0.00012). Other measures at the anaerobic threshold, respiratory compensation point, or maximal parameters remained stable without significant differences. The precise results of pre- and post-infection measurements have been shown in Table 2. After adjusting HR for training experience particular groups did not differ significantly, except 6–10 years of training (*p* = 0.03–0.37 for AT and 0.02–0.46 for RCP). Moreover, VO_2_ did not differ significantly in any subgroup in model adjusted for training experience (*p* = 0.25–0.41 for AT, *p* = 0.19–0.49 for RCP and *p* = 0.19–0.46 for max).

**TABLE 2:** Differences between pre- and post- COVID-19 CPET for study population.

Variable	Pre-COVID-19	Post-COVID-19	p-value
VO_2AT_	**34.96 ± 6.49**	**32.35 ± 5.99**	**<0.00001**
VO_2ATa_	**2,650.00 ± 470.90**	**2,446.10 ± 400.30**	**<0.00001**
HR_AT_	**145.10 ± 10.90**	**141.10 ± 10.10**	**0.00140**
VE_AT_	70.80 ± 18.70	68.10 ± 14.70	0.08950
VO_2RCP_	**43.90 ± 7.40**	**40.50 ± 6.70**	**<0.00001**
VO_2RCPa_	**3,324.30 ± 512.90**	**3,063.70 ± 440.10**	**<0.00001**
HR_RCP_	**168.80 ± 9.20**	**165.10 ± 9.80**	**0.00011**
VE_RCP_	**106.80 ± 21.70**	**98.90 ± 18.30**	**<0.00001**
Lac_RCP_	**4.88 ± 1.370**	**4.34 ± 1.14**	**0.01250**
VO_2max_	**47.81 ± 8.00**	**44.97 ± 7.07**	**0.00012**
VO_2maxa_	**3,623.47 ± 552.12**	**3,406.00 ± 474.46**	**<0.00001**
HR_max_	180.80 ± 10.08	179.84 ± 9.96	0.27330
VE_max_	142.99 ± 26.89	138.50 ± 23.93	0.06830
Lac_max_	9.71 ± 2.30	9.64 ± 2.41	0.87950

Abbreviations: COVID-19, coronavirus disease 2019; VO_2AT_, oxygen uptake at the anaerobic threshold (ml·kg·min^-1^); VO_2ATa_, absolute oxygen uptake at the anaerobic threshold (ml·min^-1^); HR_AT_, heart rate at the anaerobic threshold (beats·min^-1^); VE_AT_, pulmonary ventilation at the anaerobic threshold (l·min^-1^); VO_2RCP_, oxygen uptake at the respiratory compensation point (ml·kg·min^-1^); VO_2RCPa_ absolute oxygen uptake at the respiratory compensation point (ml·min^-1^); HR_RCP_, heart rate at the respiratory compensation point (beats·min^-1^); VE_RCP_ pulmonary ventilation at the respiratory compensation point (l·min^-1^); Lac_RCP_, blood lactate concentration at the respiratory compensation point (mmol·L^-1^); VO_2max_, maximal oxygen uptake (ml·kg·min^-1^); VO_2maxa_, absolute maximal oxygen uptake (ml·kg·min^-1^); HR_max_, maximal heart rate (beats·min^-1^); VE_max_, maximal pulmonary ventilation (l min^-1^); Lac_max_, maximal blood lactate concentration (mmol·L^-1^). Data are presented as means with standard derivation (±). Significant values (*p* < 0.05) were bolded.

### CPET results

Treadmill and cycle ergometer CPET revealed additional differences. On the cycle ergometer, only absolute VO_2max_ was altered (*p* = 0.039). Although, other variables showed changes without statistical significance-speed or power at the AT (*p* = 0.066), speed or power at the RCP (*p* = 0.061), and relative VO_2max_ (*p* = 0.078). Results of CPET performed on the cycle ergometry have been shown in Table 3. On the treadmill, both relative and absolute VO_2max_ (*p* = 0.00036 and 0.00003, respectively) and speed at AT and RCP (*p* = 0.04450 and *p* = 0.00019, respectively) presented significant differences. The results of CPET performed on the cycle ergometer have been presented in Table 2, while for a treadmill in Table 4.

**TABLE 3:** Differences between pre- and post- COVID-19 CPET for cycle ergometer.

Variable	Pre-COVID-19	Post-COVID-19	p-value
Speed/power AT	162.80 ± 25.90	154.80 ± 25.90	0.066
Speed/power at RCP	245.24 ± 41.99	232.22 ± 39.71	0.061
Maximal speed/power	310.00 ± 47.15	312.22 ± 49.06	0.811
VO_2max_	45.27 ± 9.65	42.73 ± 8.19	0.078
VO_2maxa_	**3,465.65 ± 566.51**	**3,287.80 ± 466.96**	0.039
HR_max_	183.67 ± 10.67	184.00 ± 11.04	0.817
VE_max_	142.59 ± 30.98	138.05 ± 26.22	0.297

Abbreviations: COVID-19, coronavirus disease 2019; VO_2max_, maximal oxygen uptake (ml·kg·min^-1^); VO_2maxa_, absolute maximal oxygen uptake (ml·kg·min^-1^); HR_max_, maximal heart rate (beats·min^-1^); VE_max_, maximal pulmonary ventilation (l·min^-1^). Power is presented in Watts. Data are presented as means with standard derivation (±). Significant values (*p* < 0.05) were bolded. 18 athletes underwent CPET on a cycle ergometer.

**TABLE 4:** Differences between pre- and post- COVID-19 CPET for treadmill.

Variable	Pre-COVID-19	Post-COVID-19	*p*-value
Speed AT	**11.43 ± 1.42**	**11.11 ± 1.28**	**0.04450**
Speed at RCP	**14.32 ± 1.48**	**13.80 ± 1.53**	**0.00019**
Maximal speed	16.58 ± 1.57	16.39 ± 1.65	0.26400
VO_2max_	**49.29 ± 6.59**	**46.27 ± 6.10**	**0.00036**
VO_2maxa_	**3,715.11 ± 531.27**	**3,474.63 ± 472.61**	**0.00003**
HR_max_	179.13 ± 9.50	177.42 ± 8.55	0.12650
VE_max_	143.23 ± 24.76	138.88 ± 22.94	0.14230

Abbreviations: COVID-19, coronavirus disease 2019; AT, anaerobic threshold; RCP. respiratory compensation point; VO_2max_, maximal oxygen uptake (ml·kg·min^-1^); VO_2maxa_, absolute maximal oxygen uptake (ml·kg·min^-1^); HR_max_, maximal heart rate (beats·min^-1^); VE_max_, maximal pulmonary ventilation (l min^-1^). Speed is presented in km·h^-1^. Data are presented as means with standard derivation (±). Significant values (*p* < 0.05) were bolded. 31 athletes underwent CPET on a treadmill.

In further analysis, variables adjusted to time (calculated in days from COVID-19 infection to CPET) showed significance for BF, FATM, power at the AT, and HR_RCP_. Results of the time-adjusted COVID-19 consequences on CPET and physiologic variables have been presented in Tables 5, 6.

**TABLE 5:** Pearson’s r correlation coefficients for CPET and physiologic results adjusted to time.

Variable	r-Pearson	*p*-value
BF & time [days]	**0.456**	**0.001**
VO_2AT_ & time [days]	−0.146	0.317
VO_2ATa_ & time [days]	−0.107	0.466
VO_2RCP_ & time [days]	−0.258	0.073
VO_2RCPa_ & time [days]	−0.249	0.084
VO_2max_ and time [days]	−0.197	0.175
VO_2maxa_ and time [days]	−0.182	0.211
VE_RCP_ & time [days]	−0.181	0.212
HR_AT_ & time [days]	−0.165	0.258
HR_RCP_ & time [days]	**−0.301**	**0.036**
HR_max_ & time [days]	−0.244	0.091

Abbreviations: BF, body fat (%); VO_2AT_, oxygen uptake at the anaerobic threshold (ml·kg·min^-1^); VO_2ATa_, absolute oxygen uptake at the anaerobic threshold; (ml·kg·min^-1^); HR_AT_, heart rate at the anaerobic threshold (beats·min^-1^); VO_2RCP_, oxygen uptake at the respiratory compensation point (ml·kg·min^-1^); VO_2RCPa_, oxygen uptake at the respiratory compensation point (ml·min^-1^); HR_RCP_, heart rate at the respiratory compensation point (beats·min^-1^); VE_RCP_, pulmonary ventilation at the respiratory compensation point (l min^-1^); VO_2max_, maximal oxygen uptake (ml·kg·min^-1^); VO_2maxa_, absolute maximal oxygen uptake; (ml·min^-1^); HR_max_, maximal heart rate. Significant values (*p* < 0.05) were bolded.

**TABLE 6:** Spearman’s rank correlation coefficient for CPET and physiologic results adjusted to the time.

Variable	r-Spearman	*p*-value
Fat mass and time [days]	**0.331**	**0.020**
Fat-free mass and time [days]	-0.067	0.645
BMI & time [days]	0.146	0.318
Speed/power at & time [days]	**-0.288**	**0.0445**
Speed/power at RCP & time [days]	-0.241	0.095
Maximal speed/power and time [days]	-0.212	0.143
VE_AT_ & time [days]	-0.1299	0.373
VE_max_ & time [days]	-0.071	0.629
Lac_AT_ & time [days]	0.015	0.927
Lac_RCP_ & time [days]	-0.257	0.118
Lac_max_ and time [days]	-0.281	0.155

Abbreviations: BMI, body mass index (kg·m^-2^); AT, anaerobic threshold; RCP, respiratory compensation point; VE_AT_, pulmonary ventilation at the anaerobic threshold (l·min^-1^); VE_max_, maximal pulmonary ventilation (l·min^-1^); Lac_AT_, blood lactate concentration at the anaerobic threshold (mmol·L^-1^); Lac_RCP_, blood lactate concentration at the respiratory compensation point (mmol·L^-1^); Lac_max_, maximal blood lactate concentration (mmol·L^-1^). Fat mass and fat-free mass are presented in kg. Speed is presented in km·h^-1^, while power is presented in Watts. Significant values (*p* < 0.05) were bolded.

## Discussion

The harmful influence of mild COVID-19 infection on CPET performance in a group of endurance athletes was demonstrated. The main findings are: (1) COVID-19 infection causes deterioration of exercise and body anthropometric parameters both on professional and amateur EA and (2) the impact of infection was the most significant for HR and VO_2_, and (3) even the mild COVID-19 infection should be considered as a limiting factor for exercise performance. We noticed the weight and BMI increases. Interestingly, the percentage of BF and FATM is lower after contracting COVID-19.

Among our subjects, we noticed a difference in body composition before and after contracting COVID-19. Patients during the infection are at risk of weight loss due to weakness, fever, decreased appetite and taste, depending on the advancement of the disease (Yang et al., 2020). Factors that correlate with weight loss include: raised C-reactive protein levels, impaired kidney function, and long duration of illness (Anker et al., 2021). Among our patients, both weight and BMI increased, which is not typical for the immediate time of the disease. This may be due to the passage of time since the first test, as well as the lockdown and limited opportunities for physical activity and changes in eating habits such as overeating (Martinez-Ferran et al., 2020). A systematic review was created, which among respondents during the pandemic noted an increase in food consumption by 36.3%–59.6% of respondents and a decrease in physical activity by 67.4%–61.4% (Chew and Lopez, 2021). An unexpected decrease in BF and FATM with weight gain may be due to a specific diet aimed at minimizing the increase in body fat in athletes (Iraki et al., 2019) Adequate amount of protein in the diet and properly workouts support the burning of body fat and promote the retention of lean mass (Ruiz-Castellano et al., 2021).

Physical training allows one to exert higher intensities with lower lactate levels (San-Millan and Brooks, 2018) and increase VO_2max_, which constitutes the basis for preparation to achieve optimal sports results (Seiler, 2010). Maintenance of physical fitness requires adherence to the exercise and training regimen. On the contrary, conditions of the COVID-19 pandemic presented many risks of reverse effects (Jodczyk et al., 2022). He disease itself may injure multiple organ systems, the more severe it is. However, even a relatively mild course of the illness has been associated with persistent health complications (van Voorthuizen et al., 2022). It is worth emphasizing because none of our study participants was hospitalized because of COVID-19 infection. One of the studies also showed that healthy patients who experienced mild COVID-19 have reduced efficiency compared to the control group, including reduction in peak VO2 associated with impaired systemic oxygen extraction, additionally, convalescents also showed greater respiratory failure (Singh et al., 2022). Convalescents report reduced daily physical activity, which in turn may lead to a decrease in efficiency (Delbressine et al., 2021). Despite the occurrence of side effects even in people with moderate to mild COVID-19, it is important to remember that these effects are potentially less persistent than in patients with severe COVID-19 (Jimeno-Almazan et al., 2022).

Evidence of lung fibrosis, although not common (in 1.1% of cases), was found even in mild-to-moderate cases of COVID-19 (Xia et al., 2020). Autonomic nervous system dysregulation in young patients, measured by HR variability and partly attenuated by physical activity, was also reported (Freire et al., 2022). A study of college athletes revealed signs of ongoing myocarditis or earlier myocardial injury in cardiac magnetic resonance performed up to 53 days after a period of quarantine (Rajpal et al., 2021). Muscle deconditioning was proposed to mainly limit CPET results in COVID-19 survivors (Rinaldo et al., 2021). Among possible mechanisms involved are direct cell invasion, ACE2 downregulation, and inflammation, as well as prolonged bedrest and hypoxia in severe cases (Seixas et al., 2022). Deconditioning due to hypoactivity occurs rapidly, e.g., just 5 days may be enough to negatively affect muscle strength or oxidative capacity, induce fiber atrophy and disrupt protein balance (Fovet et al., 2021). Given ordinary conditions, 8 weeks of a sedentary lifestyle was found to lead to wasted 6-week training results in terms of VO_2_ (Fritzen et al., 2020). It is also noteworthy that a pandemic does not mean quarantine itself. Apart from COVID-19 illness, legal and social conditions promoted physical inactivity by encouraging social distancing and restricting access to sports facilities, sometimes even prohibiting unnecessary leaving residential buildings (Wunsch et al., 2022).

Our results demonstrate the cumulative negative effect of the pandemic on physical fitness among endurance athletes and show different infection consequences for both treadmill and cycle ergometry. These findings are in line with the previous research, as Price et al. confirmed that both modalities differed (Price et al., 2022), the choice of device was adapted to the leading discipline of the subject, differences may have occurred in VO_2_ and HR at maximum exertion, at AT and at RCP between cycling and running CPET testing, in both males and females. In our participants, we found lower VO_2max_ and earlier lactate accumulation during CPET. Meanwhile, there are few studies that demonstrate the change in CPET performance after COVID-19 infection in endurance athletes and compare them with healthy controls. Similarly, to our results, in a sample of elite cross-country skiers—asymptomatic during evaluation, 4–6 weeks after COVID-19 diagnosis—CPET revealed lower VO_2max_, VE, HR, and O_2pulse_ when compared to the control group. On the other hand, in contrast to our observations, there was no statistical difference in VO_2max_ between post-COVID-19 soccer players and controls at the age of 18–35. Although, the trend towards reduced VO_2_ was visible. It is also worth noting that we recorded a decline in VO_2_ of 2–4 mlkg·min^−1^ over about 1.5 years between CPETs (exact interval was 591.67 ± 282.24 days; see Table 1). According to reference standards provided by Kaminsky et al. (2015) VO_2_ should decrease by about 4–5 mlkg·min^−1^ over 10 years. Our EAs experienced a much more dynamic decline in VO_2_ than only based on age. Hence, it is worth considering the previous COVID-19 infection as a possible influencing variable. Resting and maximal HR, VE, and blood pressure were not changed compared to healthy controls, as well as other reported CPET parameters. The research excluded non-competitive athletes and required recovery from infection within the previous 30 days, which means earlier assessment than used in this study. Consequently, only 5 out of 24 examined post-COVID-19 soccer players showed no illness symptoms (Komici et al., 2021). Likewise, no deterioration of CPET performance was observed in elite swimmers from National Swim Team Hungary after a mild COVID-19 infection (Csulak et al., 2021). It is worth noting that physical performance declined with age to various degrees between participants, but age was also one of the contributing factors (Wiecha et al., 2022). So far, it has been postulated that training experience may reduce the decline in HR and VO_2_ observed with age (Tanaka and Matsuura, 1984; Kaminsky et al., 2015). The less trained EAs usually noted a steeper decline. In our study, two most significantly different variables before and after infection, i.e., HR (at AT and RCP) and VO_2_ (at AT, RCP and max) after adjusting for training experience did not show significant differences (except HR for subgroup with 6–10 years of training). Thus, underwent COVID-19 infection could be a contributing factor and cause a steeper deterioration of endurance capacity.

The maximal exercise capacity, formulated in metabolic equivalents of the task (MET) was linked with the risk of hospitalization due to COVID-19 with each 1 MET higher indicating odds lower by 13% (Brawner et al., 2021). In addition, age-related mortality of the disease remains in close contact with VO_2max_ declining over the course of the human lifespan. SARS-CoV-2 infection may unfavorably influence lipid metabolism pathways and cause mitochondrial dysfunction in a similar way to aging. Therefore, there could be a harmful cumulative effect on the athletes with age, as well as protection for physically fit people (Spedding et al., 2022). Despite COVID-19 infection, in our study, athletes’ muscle deconditioning due to pandemic-related lower physical activity might be enough to explain the results. What is more, lactate accumulation is known as an immunomodulatory factor because of its connection with IL-6 production, the activity of dendritic cells, or T-cell responses, which explains some of the anti-inflammatory characteristics of physical activity. Hence, it was even hypothesized that increasing the anaerobic threshold may protect against COVID-19 complications (AbdelMassih et al., 2021) and our findings may even indicate a pro-inflammatory aspect of the pandemic.

As sports performance, presented changes in CPET parameters need to be assessed in terms of the smallest worthwhile change (SWC). Most of them decreased by about 0.4 of baseline SD. The biggest post-COVID-19 declines were observed in VO_2_ at RCP (0.46 SD) and absolute VO_2_ at RCP (0.51 SD). This constitutes a visible factor during competitions if we define SWC as 0.2 of the baseline SD (Lindberg et al., 2022). Our other results are somewhat inconclusive. Division by measurement method (cycle ergometer or treadmill) indicated some statistical differences, yet they were most likely a matter of the small size of the groups distinguished. Besides, the time-dependence of BF percentage and FATM or power at the AT and HR at the RCP cannot be discriminated among the infection, detraining, and the passage of time.

### Practical and clinical implications

Our results have practical application in both clinical circumstances and during training prescription. They provide valuable information for Medical professionals and Personal trainers. Knowing the potential consequences of mild COVID-19 will help to adjust the intensity of exercise properly to keep it safe for the athlete and select proper recovery strategies (Wisniowski et al., 2022). In addition, it will allow physicians to prescribe proper treatment or cardiac rehabilitation protocols for patients after COVID-19 (Kasiak et al., 2022b). They can also facilitate the evaluation of the effectiveness of cardiac rehabilitation programs.

### Limitations

The limitation of the study is the relatively long period between both CPET evaluations. It may contribute to some degree to changes in exercise and anthropometric data parameters. Subjects underwent CPET in different periods of the season (i.e., competition preparation time or post-season recovery period). Perhaps they could be at different fitness level at the time of CPET. Additionally, study cohort is not numerous. Above-mentioned limitations resulted from study character, which was an observational study, not a clinical trial. We are aware of the existing limitations, thus we recommend careful extrapolation of the provided results and we suggest that their highest repeatability would be seen in similar population of endurance trained individuals. Furthermore, wer recommend future research to validate our findings.

## Conclusion

A mild COVID-19 infection resulted in a decrease in professional and amateur EA’s CPET performance. The most significant changes were observed for VO_2,_ and HR. Medical Professionals and Training Specialists should be aware of the consequences of a mild COVID-19 infection in order to recommend optimal therapeutic methods and properly adjust the intensity of training. The results are a valuable addition to the current state of knowledge when preparing Return to Play clinical protocols and guidelines.

## Data Availability

The raw data supporting the conclusions of this article will be made available by the authors, without undue reservation.
